# Supplementary material for: Resolving the full spectrum of human genome variation using Linked-Reads
Source: Genome Res. 2019 Apr;29(4):635–45. doi: 10.1101/gr.234443.118 (PMC6442396; doi:10.1101/gr.234443.118)
Supplement: Supplemental Material [file supp_gr.234443.118_Supplemental_longranger-2.2.zip › longranger-2.2.tar.bz2!/lr2.2-public/lib/stan/cmdstan/stan/doc/api/html/compiler_8hpp_source.html]

Stan: src/stan/gm/compiler.hpp Source File


|  |  |
| --- | --- |
|  | Stan  2.5.0  probability, sampling & optimization |


- Main Page
- Related Pages
- Namespaces
- Classes
- Files

- File List
- File Members

All Classes Namespaces Files Functions Variables Typedefs Enumerations Enumerator Friends Macros Pages

- src
- stan
- gm

compiler.hpp

Go to the documentation of this file.

1 #ifndef STAN\_\_GM\_\_COMPILER\_HPP

2 #define STAN\_\_GM\_\_COMPILER\_HPP

3

4 #include <stan/gm/ast.hpp>

5 #include <stan/gm/generator.hpp>

6 #include <stan/gm/parser.hpp>

7

8 #include <iostream>

9 #include <string>

10

11 namespace stan {

12

13  namespace gm {

14

37  bool compile(std::ostream\* msgs, // for warnings

38  std::istream& stan\_gm\_in,

39  std::ostream& cpp\_out,

40  const std::string& model\_name,

41  const std::string& in\_file\_name = "input") {

42  program prog;

43  bool parsed\_ok = parse(msgs,stan\_gm\_in,in\_file\_name,model\_name,prog);

44  if (!parsed\_ok)

45  return false; // syntax error in program

46  generate\_cpp(prog,model\_name,cpp\_out);

47  return true;

48  }

49

50

51  }

52

53 }

54

55 #endif

stan::gm::parse

bool parse(std::ostream \*output\_stream, std::istream &input, const std::string &filename, const std::string &model\_name, program &result)

**Definition:** parser.hpp:61

stan::gm::program

**Definition:** ast.hpp:795

generator.hpp

stan::gm::generate\_cpp

void generate\_cpp(const program &prog, const std::string &model\_name, std::ostream &out)

**Definition:** generator.hpp:4614

parser.hpp

ast.hpp

stan::gm::compile

bool compile(std::ostream \*msgs, std::istream &stan\_gm\_in, std::ostream &cpp\_out, const std::string &model\_name, const std::string &in\_file\_name="input")

Read a Stan model specification from the specified input, parse it, and write the C++ code for it to ...

**Definition:** compiler.hpp:37


---

[ Stan Home Page ]

*© 2011–2014,
Stan Development Team.*
